# Characterization of neural stem cells derived from human stem cells from the apical papilla undergoing three-dimensional neurosphere induction

**DOI:** 10.1590/1678-7757-2023-0209

**Published:** 2023-11-01

**Authors:** Anupong Thongklam SONGSAAD, Sarut THAIRAT, Peeratchai SEEMAUNG, Amarin THONGSUK, Tatcha BALIT, Nisarat RUANGSAWASDI, Chareerut PHRUKSANIYOM, Thanasup GONMANEE, Kenneth L. WHITE, Charoensri THONABULSOMBAT

**Affiliations:** 1 Mahidol University Faculty of Dentistry Department of Anatomy Bangkok Thailand Mahidol University, Faculty of Dentistry, Department of Anatomy, Bangkok, Thailand.; 2 Mahidol University Faculty of Dentistry Oral Tissues, Cells and Molecular Biology Analysis and Research Center Bangkok Thailand Mahidol University, Faculty of Dentistry, Oral Tissues, Cells and Molecular Biology Analysis and Research Center, Bangkok, Thailand.; 3 Mahidol University Faculty of Science Department of Anatomy Bangkok Thailand Mahidol University, Faculty of Science, Department of Anatomy, Bangkok, Thailand.; 4 Mahidol University Faculty of Dentistry Department of Pharmacology Bangkok Thailand Mahidol University, Faculty of Dentistry, Department of Pharmacology, Bangkok, Thailand.; 5 Mahidol University Faculty of Medicine Ramathibodi Hospital Chakri Naruebodindra Medical Institute Samut Prakan Thailand Mahidol University, Faculty of Medicine Ramathibodi Hospital, Chakri Naruebodindra Medical Institute, Samut Prakan, Thailand.; 6 Utah State University College of Agriculture and Applied Sciences Department of Animal, Dairy, and Veterinary Sciences Utah The United States of America Utah State University, College of Agriculture and Applied Sciences, Department of Animal, Dairy, and Veterinary Sciences, Utah, The United States of America.

**Keywords:** Mesenchymal stem cells, Progenitor cells, Neuronal differentiation

## Abstract

**Objectives:**

The endogenous repairing based on the activation of neural stem cells (NSCs) is impaired by neurodegenerative diseases. The present study aims to characterize human stem cells from the apical papilla (hSCAPs) with features of mesenchymal stem cells (MSCs) and to demonstrate the neuronal differentiation of hSCAPs into NSCs through the formation of three-dimensional (3D) neurospheres, verifying the structural, immunophenotyping, self-renewal, gene expression and neuronal activities of these cells to help further improve NSCs transplantation.

**Methodology:**

The hSCAPs were isolated from healthy impacted human third molar teeth and characterized as MSCs. They were then induced into 3D-neurospheres using a specific neural induction medium. Subsequently, the intra-neurospheral cells were confirmed to be NSCs by the identification of Nissl substance and the analysis of immunofluorescence staining, self-renewal ability, and gene expression of the cells. Moreover, the neuronal activity was investigated using intracellular calcium oscillation.

**Results:**

The isolated cells from the human apical papilla expressed many markers of MSCs, such as self-renewal ability and multilineage differentiation. These cells were thus characterized as MSCs, specifically as hSCAPs. The neurospheres induced from hSCAPs exhibited a 3D-floating spheroidal shape and larger neurospheres, and consisted of a heterogeneous population of intra-neurospheral cells. Further investigation showed that these intra-neurospheral cells had Nissl body staining and also expressed both Nestin and SOX2. They presented a self-renewal ability as well, which was observed after their disaggregation. Their gene expression profiling also exhibited a significant amount of NSC markers (*NES*, *SOX1*, and *PAX6*). Lastly, a large and dynamic change of the fluorescent signal that indicated calcium ions (Ca^2+^) was detected in the intracellular calcium oscillation, which indicated the neuronal activity of NSCs-derived hSCAPs.

**Conclusions:**

The hSCAPs exhibited properties of MSCs and could differentiate into NSCs under 3D-neurosphere generation. The present findings suggest that NSCs-derived hSCAPs may be used as an alternative candidates for cell-based therapy, which uses stem cell transplantation to further treat neurodegenerative diseases.

## Introduction

Neurological disorders affecting the central nervous system (CNS) were cited by the Global Burden of Disease Study and lead to permanent disability and mortality. These disorders result from abnormalities in bodily structures and biochemical or physiological properties, which cause the loss of functional neurons.^1^ They highly affect neural stem cells (NSCs), neural progenitor cells (NPCs), and the adult neurogenesis ability.^2^ Unfortunately, the endogenous repair of tissue through progenitor cells is limited to 2 active regions: the subventricular zone (SVZ) and the subgranular zone (SGZ).^3^ There is, however, an alternative treatment to neuronal regeneration: modified-exogenous NSCs transplantation.^4^ Although this procedure is promising, the use of NSCs from the fetal brain, adult brain, or high potency-embryonic stem cells (ESCs) results in donor site morbidity and generates ethical concerns.^5^

Researchers have been working on techniques to make this treatment ethical, such as the characterization of human stem cells from the apical papilla (hSCAPs) of developing teeth as MSCs.^6^ These hSCAPs can differentiate into specialized cells under optimal induction environments, such as those of adipogenic, osteogenic, and neurogenic lineages.^7^ The hSCAPs have demonstrated their potential for neuronal differentiation through the expression of neuronal-associated markers.^8^ These cells are ectomesenchymal stem cells. They originate from migratory neural crest stem cells and present a superior and committed neuronal differentiation ability.^9^ Moreover, the hSCAPs can be obtained from dental waste through non-invasive procedures, which means they are easily accessible.^10^

The formation of neurospheres is a functional approach that provides a three-dimensional (3D) microenvironment for the differentiation of NSCs.^11^ Establishing the optimal conditions for the induction of neurospheres requires specific growth factors, such as the basis fibroblast growth factor (bFGF) and the epidermal growth factor (EGF),^12^ an appropriate induction period and unique cell culture vessels that trigger the formation of NSCs.^13^ Interestingly, 3D-neural induction shows superior neuronal differentiation compared with the monolayer method.^14^ These factors indicate that hSCAPs could be excellent candidates to generate NSCs under the formation of 3D-neurospheres, allowing medical professionals to overcome the limitations of modified-exogenous NSC transplantation.

The present study aims to characterize hSCAPs with properties of MSCs, then to differentiate them into NSCs through the generation of 3D-neurospheres and to further verify their structural, immunophenotyping, self-renewal, gene expression, and neuronal activities to identify the potential of this approach for the treatment of neurodegenerative diseases.

## Methodology

### Collection of tooth samples

We obtained human impacted third molars from three Thai patients aged 15-20 years who visited the Oral and Maxillofacial Surgery Clinic at the Dental Hospital of Mahidol University in Thailand. A careful selection was performed, and only the teeth with healthy apical papilla tissue and no signs of caries, pulp necrosis, trauma, or periodontal disease were used in this study. The present experimental procedures were approved by the Ethics Committee on Human Rights Related to Human Experimentation of the Faculties of Dentistry and Pharmacy at Mahidol University, Thailand (approval no. MU-DT/PY-IRB 2021/016.0706), and written informed consent was obtained from all participants before their inclusion in the study. The present authors ensured that all the procedures were performed in accordance with the Declaration of Helsinki.

### Cell detection and culture

A dual enzymatic digestion method was used to detect hSCAPs, as previously described.^7,9^ The teeth were first placed in a proliferation medium consisting of Minimum Essential Medium (MEM, Gibco, Life Technologies, Grand Island, NY, USA) supplemented with 10% fetal bovine serum (FBS, Gibco, Life Technologies) and 1% antibiotic-antimycotic (Gibco, Life Technologies). They were then washed with 0.1 M phosphate buffer saline (PBS, Sigma-Aldrich, St. Louis, MO, USA). Afterward, the apical papilla tissue was dissected and digested twice in 3 mg/mL collagenase I (Worthington, Lakewood, NJ, USA) and 4 mg/mL dispase II (Sigma-Aldrich) at 37°C for 60 minutes. The sample was filtered through a 70 µm cell strainer (Falcon^TM^, Thermo Fisher Scientific, Waltham, MA, USA), seeded into a cell culture vessel (T-75 cm^2^ flask, Nunc^TM^, Thermo Scientific, Waltham, MA, USA) and cultured in a proliferation medium in an incubator at 37°C with 5% CO_2_ and 95% humidity. The proliferation medium was changed every 2 days until an 80% confluence rate was reached. Sub-culturing by trypsinization with 0.05% trypsin-ethylenediaminetetraacetic acid (EDTA) (Gibco, Life Technologies) was then performed.^15^The Compact Cell Culture Microscope CKX3 (Olympus, Hamburg, Germany) was used to observe cell morphology and assess plastic-adherence ability.

### Cells derived from migratory neural crest stem cells

The uncharacterized cells at passage 3 were seeded in 24-well plates (Nunc™, Thermo Fisher Scientific) at a density of 2x10^4^ cells/well and cultured in the proliferation medium until they reached an 80% confluence rate. They were confirmed to be derivative of migratory neural crest stem cells through the immunocytochemistry staining of β-III tubulin and Nestin.

### Adipogenic differentiation

The uncharacterized cells at passage 3 were seeded in 24-well plates at a density of 2x10^4^ cells/well and cultured in the proliferation medium until they reached an 80% confluence rate. They were induced into adipogenic differentiation after being cultured for 4 weeks in an adipogenic induction medium consisting of MEM, 10% FBS, 1% antibiotic-antimycotic, 1 µM dexamethasone (Sigma-Aldrich), 50 µM Indomethacin (Sigma-Aldrich), 500 µM 3-isobutyl-1-methylxanthine (Sigma-Aldrich), and 1 µg/mL insulin (Sigma-Aldrich). The adipogenic induction medium was changed every 2 days. Lipid droplets were verified with Oil Red O staining, as previously described.^7^ The culture medium was then discarded and the cells were washed with PBS and fixed with 4% paraformaldehyde (Sigma-Aldrich) for 30 minutes. They were stained with 0.5 % of Oil Red O (Sigma-Aldrich) in an isopropanol (EMSURE^®^, MERCK, Darmstadt, Germany) solution for 60 minutes at room temperature, then rinsed three times in deionized water (DI H_2_O). The lipid droplet ensembles were stained and observed under the Compact Cell Culture Microscope CKX3.

### Osteogenic differentiation

The uncharacterized cells at passage 3 were seeded in 24-well plates at a density of 2x10^4^ cells/well and cultured in the proliferation medium until they reached an 80% confluence rate. They were induced into osteogenic differentiation after being cultured for 4 weeks in an osteogenic induction medium consisting of MEM, 10% FBS, 1% antibiotic-antimycotic, 50 µg/mL ascorbate-2-phosphate (Sigma-Aldrich), 0.1 µM dexamethasone, and 10 mM β-glycerophosphate (Sigma-Aldrich). The osteogenic induction medium was changed every 2 days. The calcification of an extracellular matrix was observed with an Alizarin red staining solution, used as previously described.^7^ The cultured medium was then quickly removed and the cells were washed with PBS and fixed with 4% paraformaldehyde for 30 minutes. They were stained with 40 mM of an Alizarin red (Sigma-Aldrich) solution at room temperature for 20 minutes, then rinsed three times with DI H_2_O. The extracellular matrix calcification was observed under the Compact Cell Culture Microscope CKX3.

### Neurogenic differentiation

The uncharacterized cells at passage 3 were seeded in 24-well plates at a density of 2x10^4^ cells/well and cultured in the proliferation medium until they reached an 80% confluence rate. The neurogenic differentiation was induced using two phases of a neuronal induction medium, described in a previous study.^7^ For 24 hours, the cells were induced into neurogenic differentiation with the phase I neuronal induction medium, which consisted of Dulbecco’s Modified Eagle Medium supplemented with Nutrient Mixture F-12 (Ham) (DMEM/F-12, Gibco, Life Technologies), 10% FBS, 1% antibiotic-antimycotic, 10 ng/mL bFGF (Gibco, Life Technologies), and 500 µM β-mercaptoethanol (Sigma-Aldrich). After this treatment, the cells were cultured for 6 hours in the phase II neuronal induction medium, which consisted of DMEM/F-12, 2% dimethyl sulfoxide (Sigma-Aldrich), 1% antibiotic-antimycotic and 100 µM butylated hydroxyanisole (Sigma-Aldrich). The Nissl substance of a typical neuronal cell marker was stained with Cresyl violet and observed under the Compact Cell Culture Microscope CKX3.

### Analysis of cell-surface antigen molecules

The uncharacterized cells at passage 3 were seeded in a T-75 cm^2^ flask at a density of 1x10^6^ cells and cultured in the proliferation medium until they reached an 80% confluence rate. Subsequently, cells at the density of 5x10^5^ cells were harvested and had their surfaces analyzed to assess the presence and profile of antigen molecules. This analysis was conducted with the BD FACSMelody™ Cell sorter (BD Biosciences, San Jose, CA, USA). Five specific antibodies were used as markers for MSCs properties, including the APC/Cy7-conjugated anti-human CD73 (1: 100; Biolegend, San Diego, CA, USA), the PE-conjugated anti-human CD90 (1: 100; Biolegend), the Alexa Flour^®^ 488-conjugated anti-human CD105 (1: 100; Biolegend), and the FITC-conjugated anti-human CD146 (1: 100; Biolegend). The APC-conjugated anti-human CD34 (1: 100; Biolegend), which served as the marker of hematopoietic stem cells, was used as the negative control. The cell pellet was held in 0.1 M PBS-EDTA (Sigma-Aldrich) and used as an unstained compensation. The percentage of the cell-surface antigen molecule markers profiling was counted at 20,000 events and analyzed with the FlowJo™ software (BD Biosciences).

### Colony-forming unit fibroblast

The uncharacterized cells at passage 3 were seeded in 6-well plates (Nunc™, Thermo Fisher Scientific) at a density of 500 cells/well and cultured in the proliferation medium for 7 days. The medium was changed every 2 days. The colonies of these cells were observed using Giemsa staining, as previously described.^7^ The culture medium was quickly discarded and the cells underwent PBS washing and fixation with 4% paraformaldehyde in PBS for 30 minutes. They were fixed with methanol (EMSURE^®^, MERCK) for 10 minutes and washed with distilled water. Then, 1% of Giemsa solution (Sigma-Aldrich) was incubated at room temperature for 30 minutes and washed several times with PBS, until the excess staining was fully removed. The purple colonies of these cells were observed under the Compact Cell Culture Microscope CKX3.

### Neurosphere induction

To generate neurospheres, the characterized hSCAPs at passage 5 were induced at a density of 6.25x10^4^ cells/well and cultured in a neural induction medium composed of DMEM/F-12 supplemented with 2% B-27 (Gibco, Life Technologies), 20 ng/mL EGF (Gibco, Life Technologies), 20 ng/mL bFGF and 1% antibiotic-antimycotic, placed in a Costar^®^ ultra-low attachment multiple-well plate, size 24 well (Corning, NY, USA). The neurospheres were kept in the neural induction medium for 5 days. Half of the medium was changed every two days. The morphology of the neurospheres was observed and imaged with the Compact Cell Culture Microscope CKX3. Furthermore, the properties of NSCs in the neurospheres were detected through the identification of Nissl substance, made with Cresyl violet staining, the protein expression evaluation, made with immunocytochemistry, the analyses of self-renewal abilities and gene expressions through the reverse transcription-quantitative polymerase chain reaction (RT-qPCR), and the analysis of functional activity through intracellular calcium oscillation.

### Cresyl violet staining

The neuronal cells derived from hSCAPs, the characterized hSCAPs, and the neurospheres were fixed in 4% paraformaldehyde at room temperature for 1 hour, then washed with PBS for 5 minutes and with double distilled water (ddH_2_O) for 1 minute. Subsequently, they were incubated with a Cresyl Violet Acetate working solution (Electron Microscopy Sciences, Hatfield, PA, USA) in a dark environment for 1 hour. Thereafter, the three specimens were washed with ddH_2_O, then with 90, 95, and 100% ethanol (EMSURE^®^, MERCK), respectively (according to the order first cited in this section). The stained Nissl substance was observed under the Compact Cell Culture Microscope CKX3.

### Immunocytochemistry

The uncharacterized hSCAPs, the characterized hSCAPs, and the neurospheres were fixed in 4% paraformaldehyde (in PBS) at room temperature for 1 hour. Subsequently, they were kept in PBS and 20% cold-methanol (in PBS) at room temperature, then washed with PBS again. Thereafter, the specimens were permeabilized with 0.5% Triton X-100 (Sigma-Aldrich) (in PBS) at 4°C overnight and blocked with 15% bovine serum albumin (BSA, Sigma-Aldrich) at 4°C for 12 hours. They were moistly incubated at 4°C for 24 hours with the following primary antibodies: mouse anti-β-III tubulin (1:1,000; Biolegend), mouse anti-Nestin (1:500; Biolegend), and rabbit anti-SOX2 (1:500; Abcam, Cambridge, UK). All of the antibodies were diluted with 5% BSA (in PBS with 0.05% Tween-20 [Sigma-Aldrich]). After this primary incubation, the cells were incubated at room temperature for 4 hours with the following secondary antibodies: Goat anti-mouse Alexa Flour plus 488 (1:1,000; Invitrogen; Thermo Fisher Scientific, Waltham, MA, USA) and donkey anti-rabbit Alexa Flour plus 594 (1:1,000; Invitrogen). Nuclei were counterstained and mounted with the Prolong™ Diamond antifade mountant with 4′,6-diamidino-2-phenylindole (DAPI; Invitrogen). The samples were observed under the Confocal Microscope Platforms STELLARIS 5 (Leica Microsystems, Wetzlar, Germany) and imaged with the Leica Application Suite X software (Leica microsystems). The fluorescent intensity in the cells was measured using the ImageJ software (NIH, Bethesda, MD, USA).

### Self-renewal ability

To demonstrate the self-renewal ability of these NSCs, the neurospheres at passage 1 were collected and enzymatically dissociated with Accutase (Gibco, Life Technologies) for 3 minutes, in an incubator at 37°C with 5% CO_2_ and 95% humidity. Subsequently, the dissociated intra-neurospheral cells were resuspended with the neural induction medium, seeded in the Costar^®^ ultra-low attachment multiple well-plate, size 24 well, and kept inside it. Half of the medium was changed every two days. After 5 days, the morphology of neurospheres at passage 2 was observed under the Compact Cell Culture Microscope CKX3.

### RT-qPCR

**Figure 1:**
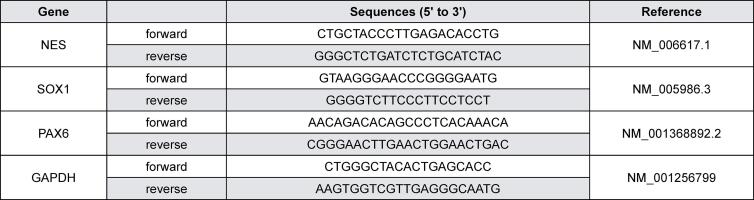
Primer sequences for RT-qPCR

The characterized hSCAPs at passage 4 and the neurospheres at passage 1 were collected, lysed and had their total RNA extracted with a High Pure RNA Isolation Kit (Roche, Basel, Switzerland). The RNA was quantified using the Nanodrop™ 2000/2000c spectrophotometers (Thermo Scientific). The extracted RNA was reverse-transcribed into cDNA with the Transcriptor First Stand cDNA Synthesis Kit (Roche) and qPCR was performed with the CFX96 real-time PCR Detection System (Bio-Rad, Hercules, CA, USA) using the KAPA SYBR FAST qPCR kits (Sigma-Aldrich). The thermocycling conditions for the qPCR were: 95°C for 180 seconds, followed by 40 cycles of 95°C for 3 seconds and of 60°C for 30 seconds. The primer pairs used for qPCR (Integrated DNA Technologies, the Gemini Singapore Science Park II, Singapore) are listed in Figure 1. The gene expression level was calculated using the 2^-^ method.^16^

### Intracellular calcium oscillation

The intracellular calcium influx of the neurospheres was evaluated to identify their potential neuronal activity. The intracellular calcium influx was assessed in accordance with descriptions by previous studies.^7,17^ The neurospheres at passage 1 were incubated with DMEM/F-12, 1% antibiotic-antimycotic, 0.08% pluronic acid (Invitrogen), and 3 µM Fluo-3 AM (Invitrogen), which is a fluorescence chelator of intracellular calcium (Ca^2^), at 37°C with 5% CO_2_ and 95% humidity for 60 minutes. The neurospheres were subsequently washed with DMEM/F-12, 1% antibiotic-antimycotic, and PBS, then immediately put into Tyrode’s solution (1 mM MgCl_2_, 2 mM CaCl_2_, 5 mM KCl, 25 mM HEPES, 30 mM glucose and 129 mM NaCl, pH 7.4; all from Sigma-Aldrich) and kept inside it. The characterized hSCAPs at passage 4 were used as a negative control. The neuronal activity was triggered with 50 mM KCI. Subsequently, the fluorescent intensity was recorded at excitation 506 nm for 120 seconds, with the Confocal Microscope Platforms STELLARIS 5, and the mean intensity of the fluorescent signals was assessed using the Leica Application Suite X software, which allowed for the interpretation of the neuronal activity.

### Statistical analysis

Data are expressed as the mean ± standard deviation (SD) of the three experimental replicates. Different groups were compared by the unpaired Student’s t-test, conducted by the GraphPad Prism (San Diego, CA, USA). The differences in which *p<0.05 were considered statistically significant.

## Results

### Characterization of hSCAPs

**Figure 2:**
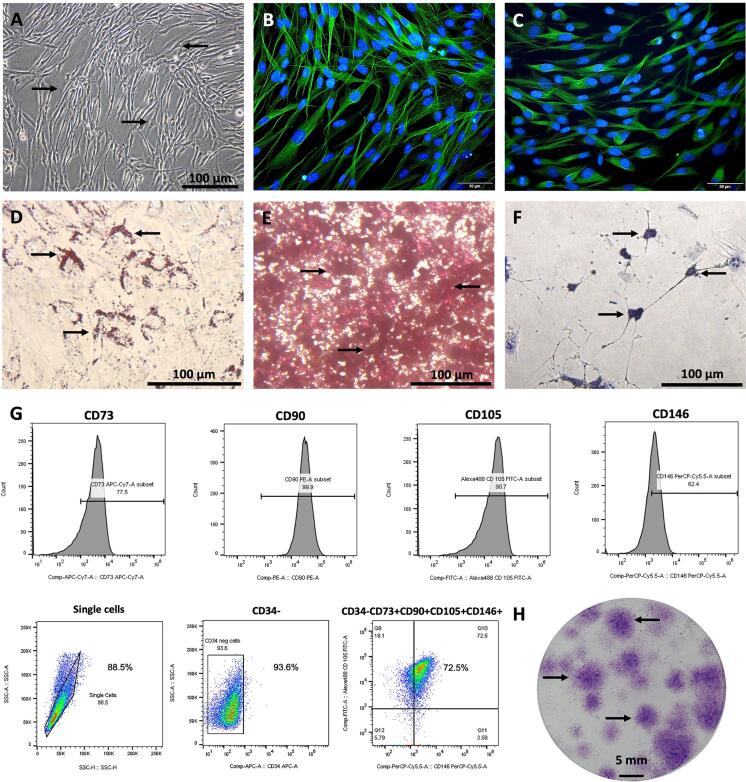
Characterization of hSCAPs. (a) The isolated cells can grow on plastic adherent culture vessels and reveal the typical fibroblast-like shape morphology. (b-c) The neural crest stem cells' derivative origin was demonstrated with β-III tubulin and nestin staining, respectively. (d) The number of isolated cells that expressed these markers (CD34−, CD73+, CD90+, CD105+, and CD146) are highly expressed. (e) The isolated cells can form colonies. (f-h) Multipotential differentiation abilities were demonstrated by adipogenesis, osteogenesis, and neurogenesis, respectively. Scale bars: a, f, g, and h = 100 µm, b and c = 50 µm, and e = 5 mm

An analysis was conducted to determine whether the cells isolated from human apical papilla tissues exhibited characteristics commonly associated with MSCs. The cells had a fibroblast-like shape and could grow on plastic-adherent culture vessels (Figure 2A). The immunofluorescence staining confirmed that the cells were derivative of migratory neural crest stem cells, as they positively stained for β-III tubulin (Figure 2B) and Nestin (Figure 2C). Moreover, under appropriate differentiation-inducing conditions, the cells differentiated into adipocytes, osteocytes, and neuronal cells: they presented accumulated lipid droplets (Figure 2D), secreted calcified nodules (Figure 2E), and exhibited Nissl substance (Figure 2F), as revealed through Oil Red O, Alizarin red, and Cresyl violet staining, respectively. Moreover, the flow cytometry profiling of the cell-surface antigen molecules in these cells demonstrated positive markers for MSCs, including CD73, CD90, CD105, and CD146, as indicated by the high intensity of histograms. A great portion of the cells did not express CD34 and co-expressed CD34-, CD73+, CD90+, CD105+, and CD146+ (Figure 1G). Colonies of isolated cells with positively stained Giemsa dye, used to evaluate self-renewal properties, were formed (Figure 1H). Taken together, the isolated cells derived from human apical papilla tissue exhibited properties of MSCs and were verified to be hSCAPs.

### Generation of neurospheres

**Figure 3:**
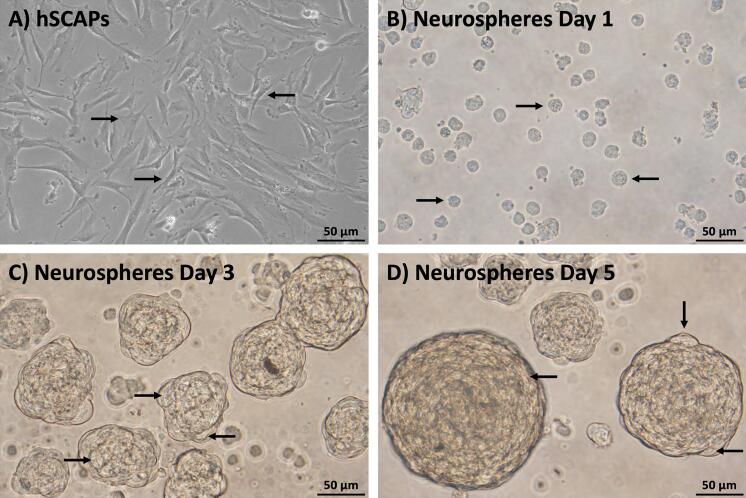
Generation of neurospheres. (a) The hSCAPs presented the typical fibroblast-like shape. (b) Neurospheres exhibited free-floating aggregated cells and consisted of a cluster of intra-neurospheral cells. Scale bars: a and b = 100 µm

Placed inside an ultra-low attachment multiple-well plate, regularly filled with a neutral induction medium, the characterized hSCAPs changed morphology, transforming themselves, on day 1, from typical fibroblast-like shapes (Figure 3A) into free-floating cells (Figure 3B). These cells then aggregated, forming 3D-spheroid clusters known as ‘neurospheres’. The size of the neurospheres increased in a time-dependent manner on days 3 (Figure 3C) and 5 (Figure 3D). These neurospheres consisted of intra-neurospheral cells with NSC properties, which were subsequently characterized.

### Identification of Nissl substance

**Figure 4:**
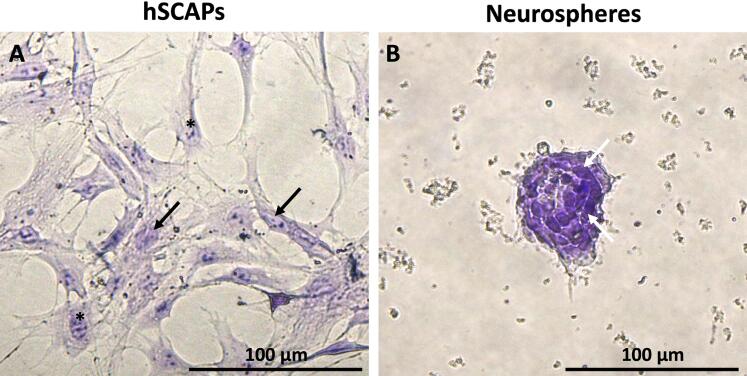
Identification of Nissl substance. (a) The hSCAPs revealed the pale purple background of the nucleus (black arrows) and the violet spot of the nucleolus (black asterisks). (b) The cluster of intra-neurospheral cells exhibited an intense purple substance (white arrows) that indicates the Nissl body of neuronal cells marker. Scale bars: a and b = 100 µm

The presence of the Nissl body, a typical neuronal substance, was confirmed by the Cresyl violet staining. The hSCAPs presented a typical fibroblast-like shape morphology, a pale purple background, characteristic of a nucleus (black arrows), and a dark spot, typical of a nucleolus (black asterisks) (Figure 4A). Interestingly, the cell body in the cluster of intra-neurospheral cells presented an intense purple substance (white arrows), which indicated typical neuronal substance (Figure 4B) and was consistent with the neuronal cells derived from hSCAPs under the two-dimensional neuronal differentiation (Figure 4F). These results suggest that the neurospheres consisted of neuronal cells.

### Immunofluorescence phenotyping of NSCs

**Figure 5:**
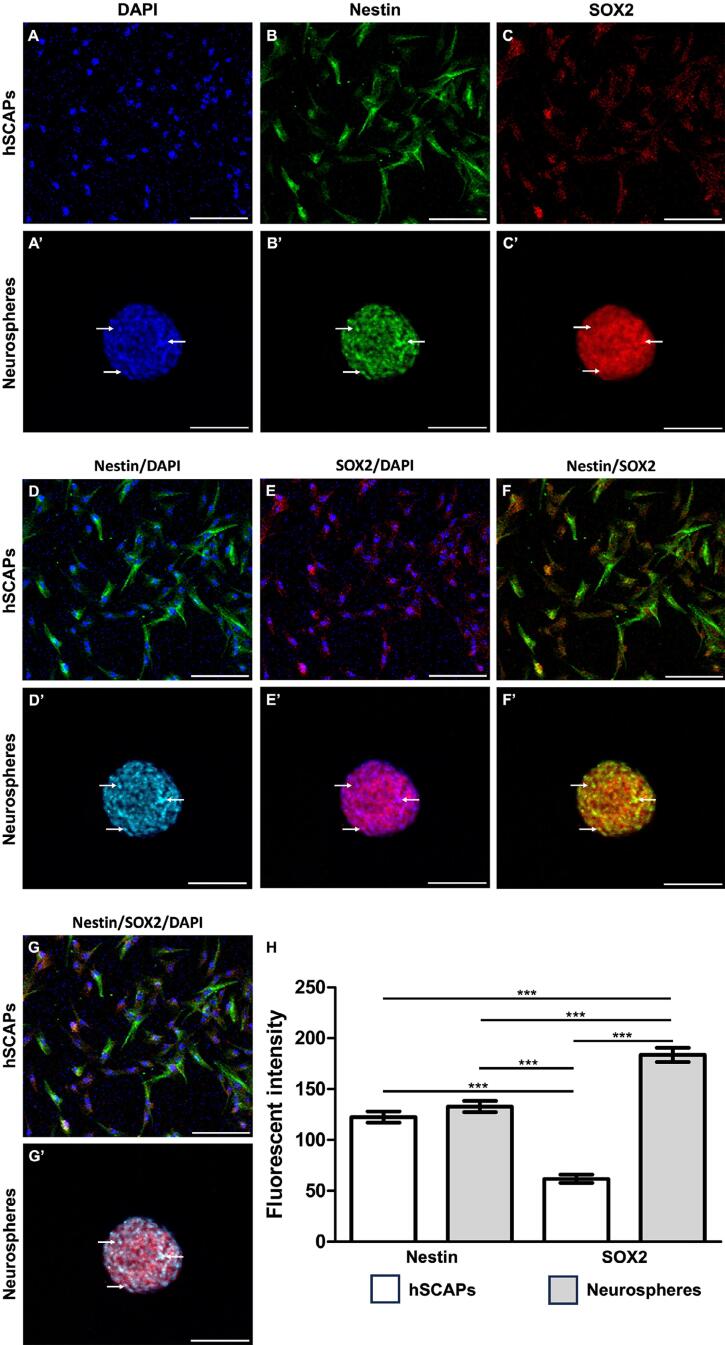
Immunofluorescence phenotyping of NSCs. (a-c) Single immunofluorescences profiling including DAPI, nestin, and SOX2, respectively. (d-f ) Double immunofluorescences profiling. (g) The intra-neurospheral cells were co-positively expressed and localized nuclei markers which were characterized as NSCs. Scale bars: a-g = 100 µm

To have their NSC properties assessed, neurospheres underwent an immunofluorescence staining of their NSC markers and were compared with the characterized hSCAPs. First, the characterized hSCAPs were stained with DAPI to have their nuclei located (Figure 5A). In the hSCAPs, the expression of Nestin (Figure 5B), which indicated ectomesenchyme origins, along with the presence of SOX2 (Figure 5C) indicated that these cells had pluripotency. Interestingly, the individual intra-neurospheral cells presented their nuclei after undergoing DAPI staining (Figure 5A’) and also expressed both Nestin (Figure 5B’) and SOX2 (Figure 5C’), which represented the specific NSC markers. Double immunofluorescence staining (Nestin/SOX2) was performed on hSCAPs and neurospheres to verify the characteristics they shared with NSCs (Figure 5D-G and 5D’-G’). The results demonstrated that while hSCAPs rarely co-expressed Nestin and SOX2 (Figure 5F), neurospheres did co-express the two (Figure 5F’). Furthermore, the neurospheres exhibited different staining patterns, which indicates the heterogeneity of the intra-neurospheral cell population. The individual intra-neurospheral cells, which co-expressed Nestin, SOX2, and DAPI, were confirmed to be NSCs (Figure 5G’). Lastly, the analysis of fluorescence intensity demonstrated that hSCAPs presented a higher intensity of Nestin than of SOX2. The neurospheres exhibited the greatest SOX2 expression (Figure 5H). Taken together, the present results indicate that hSCAPs can differentiate into NSCs after forming 3D neurospheres.

### Self-renewal ability

**Figure 6:**
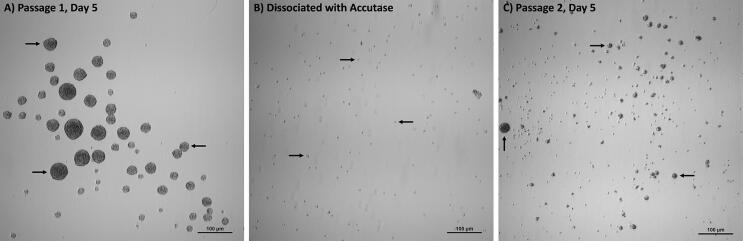
Self-renewal ability. The neurospheres can be re-formed to indicate self-renewal ability. (a) Neurospheres at passage 1. (b) dissociated neurospheres followed by Accutase. (c) Neurospheres at passage 2. Scale bars: a, b, and c = 100 µm

**Figure 7:**
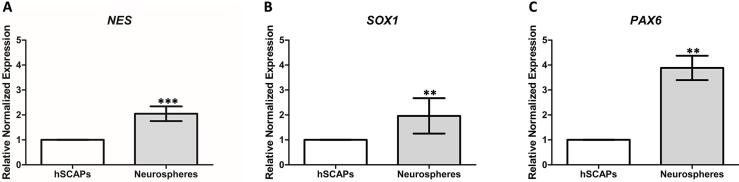
Gene profiling. (a-c) The neurospheres presented the increasing expression of NSCs profiling (<i>NES, SOX1, </i>and <i>PAX6</i>) when compared to the hSCAPs. Data were expressed as the mean ± SD; n = 3, **<i>p </i>< 0.01, ***<i>p</i> < 0.001

After undergoing neural induction for 5 days, the hSCAPs differentiated into neurospheres, which aggregated, forming a 3D-cluster of NSCs (Figure 7A). These neurospheres at passage 1 then underwent a treatment with the Accutase enzyme and disaggregated into individual cells (Figure 7B). Interestingly, the intra-neurospheral cells could be re-aggregated in the ultra-low attachment multiple-well plate and become neurospheres at passage 2, which indicates their self-renewal ability, typical of NSCs (Figure 6C).

### Gene expression profiling

At the molecular level, it was discovered that the neurospheres presented higher expressions of *NES, SOX1*, and *PAX6* than the hSCAPs. These three markers are known to indicate the presence of NSCs. This suggests that the hSCAPs differentiated into NSCs through the 3D-neurosphere induction.

### Neuronal activity

**Figure 8:**
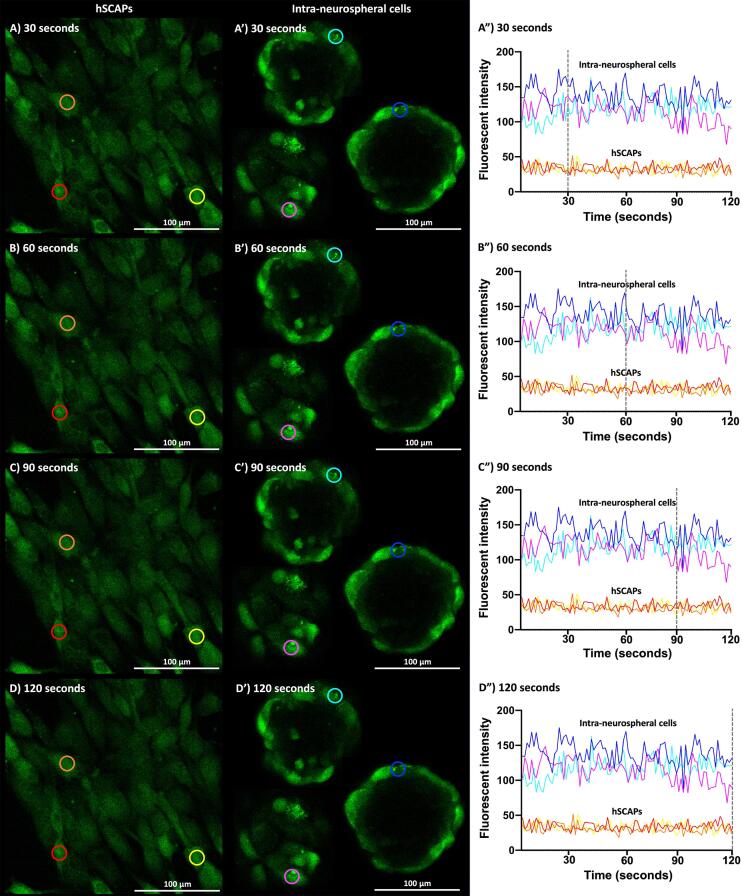
Functionality test. (a) The hSCAPs weakly expressed fluorescent calcium ions signal. (b) The intra-neurospheral cells obviously revealed calcium ions signal. (c) The hSCAPs presented a low and narrow dynamic change of calcium ions intensity (red, orange, and yellow lines). Importantly, higher and wider dynamic changes of calcium ions intensity were observed at intra-neurospheral cells (pink, dark blue, and light blue lines). Data were expressed as the mean intensity of calcium ions; n = 3

Intracellular calcium oscillation was detected in the typical neuronal cells, indicating that neuronal activity was activated by 50 mM KCI. The fluorescence intensity of Fluo-3 AM was positively correlated with the intracellular calcium concentration. Fluorescent images of the hSCAPs and intra-neurospheral cells during intracellular calcium oscillation were captured at several time points, 30, 60, 90, and 120 seconds, respectively. The hSCAPs expressed a weak fluorescent signal (Figure 8A-D) and the intra-neurospheral cells expressed an intense fluorescent signal (Figure 8A’-D’). Moreover, the intra-neurospheral cells presented dynamic changes in their fluorescence intensity, which showed high peaks and intervals (pink, dark blue, and light blue lines). In contrast, a lower, narrow dynamic change was expressed by the hSCAPs (red, orange, and yellow lines; Figure 8A’’-D’’). These results indicate that the intra-neurospheral cells presented neuronal activity.

## Discussion

Adult neurogenesis in the mammalian brain was shown to be regulated by the physiological and biological processes of NSCs, such as cell proliferation, cell differentiation, cell fate determination, cell survival, maturation, integration of the generated neuronal cells into the existing circuitry, and functional input reception.^18^ The potential compartments where NSCs could be located are the SVZ of the lateral ventricle and the SGZ of the hippocampal dentate gyrus, which are defined as the actively restricted regions for adult neurogenesis.^19^ However, their endogenous repairing by their NSCs is limited by damages in the CNS.^3^ In this study, NSCs exhibited a self-renewal ability, which resulted in their aggregation into clusters of cells^20^ that differentiated into neurons, oligodendrocytes, and astrocytes under optimal *in vitro* inducing conditions.^21^ Therefore, the transplantation of exogenous NSCs could allow for the replacement of degenerated neurons and the regeneration of injured CNSs.^4^ However, the collection of NSCs from the SVZ and SGZ of adult brains was shown to cause donor site morbidity, harming donors and creating ethical concerns.^5^ It is also complicated to isolate and cultivate the NSCs from these regions: cells can be isolated from adult brains, but at a deficient number.^22^ The present study aimed to overcome these limitations by choosing an alternative tissue to collect cells from, and resulted in the discovery of cells that could neuronally differentiate into NSCs without causing donor site morbidity and ethical concerns.

Dental-derived mesenchymal stem cells are a promising resource for neuronal regeneration processes, due to their neuronal differentiation ability.^23^Previous researches have described hSCAPs as a novel population of post-natal multipotent stem cells residing in the apical papilla tissue of immature permanent teeth.^6^ The present study analyzed stem cells located in apical papilla tissue and characterized them as MSCs in accordance with the minimal phenotypic and functional criteria established by the International Society for Cellular Therapy, which includes factors such as plastic adherence ability, cell morphology, self-renewal ability, multipotential differentiation, and the expression of cell-surface antigen molecules.^24^ The hSCAPs derived from the developing root of immature permanent teeth and consisted of a large population of early stem cells that exhibited superior properties compared with those of other adult stem cells derived from mature tissue, including the potential for differentiation and the ability to self-renew.^25^ The collection of these hSCAPs took place through a non-invasive process: the cells were easily accessible and provided by dental waste.^10^ Additionally, an *in vitro* study demonstrated that the secretome of hSCAPs has neurotrophic factors that can trigger the neurite outgrowth of human neuroblastoma cells and an *in vivo* study showed that these neurotrophic factors can enhance the regeneration of sciatic nerve injuries.^26^ The results of the present study indicate hSCAPs from dental waste as an alternative resource to generate NSCs with less ethical concerns and donor site morbidity risks.

Various strategies to differentiate cells into neurons have been recently developed, including epigenetic modification, small molecules, psychotropic drugs, and enriched medium cocktails with chemical inducers,^27^ but the placement of MSCs in a neuronal induction medium containing chemical inducers provided a faster neuronal differentiation rate than other methods.^28^ The generation of neurospheres was defined as a potential *in vitro* model for studying CNS disorders.^29^ The present study effected the neuronal differentiation of characterized hSCAPs into NSCs through a 3D neurosphere induction process, which took place within a neural induction medium containing bFGF and EGF in low-adherent culture vessels for 5 days. To form neurospheres, specimens require supplementation with specific growth factors, including bFGF and EGF, and a microenvironment suitable for neural induction and further neuronal maturation.^11,12,30^ A study found that, after undergoing a spheroid-based 3D neural induction method, NPCs derived from human-induced pluripotent stem cells (hiPSCs) expressed high concentrations of Nestin/PAX6 and differentiated into neuronal cells, exhibiting a longer neurite outgrowth than that of cells undergoing a 2D monolayer method.^31^Moreover, the formation of 3D spheres was shown to induce a higher neurogenic potential in the hSCAPs than the 2D method, resulting in overall higher neurite numbers, mean neurite lengths, total neurite lengths, and expressions of neurogenic-associated genes.^14^During the neurosphere induction process, the size of the neurospheres greatly increased, and five-day intervals generated a greater number of viable neurospheres.^13^ Furthermore, it was found that at 5 days of culture, a primary neurosphere culture originating from newborn Sprague-Dawley rats contained NSCs with a healthy morphology, but at 8 to 9 days of culture these NCSs revealed a dark area formed by dead intra-neurospheral cells.^32^

The present study investigated specific parameters and cellular structures to elucidate the characterization of *in vitro*-induced neurons.^33^ First, an analysis of cell morphology revealed that characterized hSCAPs presented typical fibroblast-like shapes, while the neurospheres they originated had a 3D-spheroidal form. This finding is similar to those of recent studies that investigated neurospheres derived from human dental pulp stem cells (hDPSCs)^12,13^ and hiPSCs.^31^ Second, it was found that the Nissl body, an intensely basophilic granular consisting of a rough endoplasmic reticulum, was only present in neurons.^34^ *In vitro*-differentiated neuronal cells derived from hSCAPs^7^ and hDPSCs^35^ were recently shown to present Nissl bodies, which were found through a staining process with Cresyl violet dye. Moreover, the hippocampal area of a rat model exhibited the organization of cells with Nissl bodies, which were characterized as neurons.^36^ Therefore, the identification of the Nissl body can be used to verify the characteristics of typical neuronal cells. In the present study, the Cresyl violet staining process revealed that the Nissl body was present in intra-neurospheral cells, which evidenced their neuronal phenotype.

Certain studies found that biomarkers for embryonic and adult neurogenesis were necessary to characterize the gene and protein expressions of NSCs.^37^ *NES*, a protein-encoding gene, was shown to encode the Nestin protein, which is expressed in diving cells during the early stages of development of the nervous system.^38^ The *SOX1* gene encodes a transcription factor that exerts an essential role in neurogenesis.^39^ *SOX2* encodes the transcription factor essential for self-renewal and is critical in maintaining NSCs.^40^ *PAX6* encodes one of the critical embryonic transcription factors, which regulates CNS morphogenesis and is widely expressed in the neuroectoderm.^41^ From the sixth to the eighth day of a neurosphere induction process, hDPSCs and human gingival mesenchymal stem cells (hGMSCs) generated NCSs that, compared with those in undifferentiated hDPSCs and hGMSCs, exhibited greater expressions of *NES* and *SOX1*, and a lower expression of *PAX6*, respectively.^42^These results imply that these induced cells presented the same NSC profile, and that a long-time neurosphere induction might affect the expression of *PAX6*. On the fifth day of the neurosphere induction process conducted in this study, neurospheres presented higher expressions of *NES, SOX1,* and *PAX6*, than the hSCAPs that were used as the negative control—and the intra-neurospheral cells were thus proven to be NSCs. Other studied found that *in vitro*-induced neurospheres derived from hDPSCs expressed Nestin,^13,17^and that neurospheres derived from stem cells of bovine adipose tissue expressed Nestin, SOX2, and β-III tubulin (neurogenic-associated protein).^43^ Moreover, it was found that *in vivo* NSCs derived from neurospheres of embryonic brain cells E14.5 to E16.5 were positive for Nestin staining, thus confirming their NSC properties.^44^ The neurospheres analyzed in the present study consisted of a heterogeneous population with different immunofluorescence staining patterns, and the co-expression of Nestin and SOX2 in the intra-neurospheral cells verified their profiling as NSC.

Subsequently in this study, the ability of the NCSs to self-renew, represented by the re-formation of the neurospheres, was demonstrated through their disaggregation with Accutase. Studies with neurospheres derived from hDPSCs found that the dissociation these clusters with Accutase allowed for the re-forming of more viable cells than the mechanical and enzymatic (trypsin) dissociations.^13^ Moreover, neurospheres generated from the primary culture of adult rat SVZ and hippocampus cells formed more new neurospheres after undergoing disaggregation with Accutase than after being dissociated with trypsin.^45^ The present study showed that a new passage of neurospheres was formed after neurospheres underwent Accutase dissociation within a neural induction medium in an ultra-low attachment culture system. These results indicate that the neurospheres presented the ability of self-renewal.

Previous studies have investigated functional neuronal networks, intercellular communication^12,46^ and intracellular signalling^17,47^ to further verify the functional profile of neuronal cells. Intracellular calcium oscillation is a technique used to investigate the status of Ca^2^ influx.^48^ Ca^2^ are essential ions that get internalized into neurons during vesicular neurotransmitter-releasing activity.^49^ The activity of intracellular calcium transients can be alternately represented as neuronal activity, which is closely correlated to electrical activity recorded with a whole-cell patch clamp.^50^ In the present study, the neuronal activity of intra-neurospheral cells was analyzed through the visualization of intracellular calcium oscillation. The Fluo-3 AM, a calcium indicator, was used to detect the activity of intracellular calcium signalling during neurotransmission.^51^ The dynamic changes in the fluorescence intensity of intra-neurospheral cells consisted of continuous high-intensity peaks and wide intervals. In contrast, steady baseline patterns of low intensity were observed in the hSCAPs used as the negative control. Since these findings on intracellular calcium oscillation were consistent with previous studies that presented the functional profilings of neuronal cells derived from hDPSCs ^17,35,52^and of hiPSCs derived from dentate gyrus neuronal progenitors,^53^ the intra-neurospheral cells analyzed in the present study were proven to be functional neuronal cells. However, further investigation with electrophysiological tests and neurogenic maturation analyses is still necessary to thoroughly characterize the neuronal profile of these cells.

The findings of this study reinforce the potential of *in vitro-*induced NSCs derivated from hSCAPs, which can be used as an alternative resource for neuronal regeneration processes, replacing animal models and reducing limitations such as donor-site morbidity and ethical concerns. The present study aimed to develop an efficient method for the production of neural commitment of hSCAPs in large-scale expansion, specifically in a serum-free medium. However, a preclinical study on the safety and efficacy of *in vitro-*induced NSCs derived from hSCAPs must be done before applying these findings to *in vivo* transplantation. Transplantation of neurospheres into damaged CNS areas should also be performed in future studies to explore factors such as host integration, cell survival, and neuronal differentiation *in vivo* models.

The results of this study demonstrated the MSC properties of hSCAPs and the neuronal profiling of NSCs derived from characterized hSCAPs that underwent a 3D neurosphere induction process. These properties were revealed through the analysis of cell morphology, protein expression (by immunofluorescence staining), self-renewal ability, gene expression, and intracellular calcium oscillation. These results suggest that NSCs derived from hSCAPs can be used for exogenous transplantation in stem cell-based therapies for neurodegenerative diseases.

## Conclusions

This study demonstrated the potential of the neuronal differentiation of hSCAPs into NSCs through the 3D-neurosphere induction process. The present findings suggest the use of NSCs derived from hSCAPs in further exogenous transplantation procedures during cell-based therapies for neurodegenerative diseases.
