# ERRATUM

**DOI:** 10.1590/1678-7757-2023er004

**Published:** 2023-12-15

The article: “**Characterization of neural stem cells derived from human stem cells from the apical papilla undergoing three-dimensional neurosphere induction**”, published at Journal of Applied Oral Science 31(e-20230209):1-16. doi: 10.1590/1678-7757-2023-0209 was published with the following errors:

**Table:** 

Page	Section	Where it reads	The sentence should read
6	Characterization of hSCAPs	… and CD146+ (Figure 1G).	… and CD146+ (Figure 2G).
6	Characterization of hSCAPs	… were formed (Figure 1H).	… were formed (Figure 2H).
6	Identification of Nissl substance	… neuronal differentiation (Figure 4F).	… neuronal differentiation (Figure 2F).
6/7	Self-renewal ability	… of NSCs (Figure 7A).	… of NSCs (Figure 6A).
7	Self-renewal ability	… individual cells (Figure 7B).	… individual cells (Figure 6B).
7	Gene expression profiling	These three markers are known to indicate the presence of NSCs.	These three markers are known to indicate the presence of NSCs (Figure 7).

Where it reads, page 7

**Figure 2-** Characterization of hSCAPs. (a) The isolated cells can grow on plastic adherent culture vessels and reveal the typical fibroblastlike shape morphology. (b-c) The neural crest stem cells' derivative origin was demonstrated with β-III tubulin and nestin staining, respectively. (d) The number of isolated cells that expressed these markers (CD34−, CD73+, CD90+, CD105+, and CD146) are highly expressed. (e) The isolated cells can form colonies. (f-h) Multipotential differentiation abilities were demonstrated by adipogenesis, osteogenesis, and neurogenesis, respectively. Scale bars: a, f, g, and h = 100 μm, b and c = 50 μm, and e = 5 mm

The sentence should read

**Figure 2-** Characterization of hSCAPs. (A) The isolated cells can grow on plastic adherent culture vessels and reveal the typical fibroblastlike shape morphology. (B-C) The neural crest stem cells' derivative origin was demonstrated with β-III tubulin and nestin staining, respectively. (G) The number of isolated cells that expressed these markers (CD34−, CD73+, CD90+, CD105+, and CD146+) are highly expressed. (H) The isolated cells can form colonies. (D-F) Multipotential differentiation abilities were demonstrated by adipogenesis, osteogenesis, and neurogenesis, respectively. Scale bars: A, D, E, and F = 100 μm, B and C = 50 μm, and H = 5 mm

Where it reads, page 9

**Figure 5-** Immunofluorescence phenotyping of NSCs. (a-c) Single immunofluorescences profiling including DAPI, nestin, and *SOX2*, respectively. (d-f ) Double immunofluorescences profiling. (g) The intra-neurospheral cells were co-positively expressed and localized nuclei markers which were characterized as NSCs. Scale bars: a-g = 100 μm

The sentence should read

**Figure 5-** Immunofluorescence phenotyping of NSCs. (A-C and A’-C’) Single immunofluorescences profiling including DAPI, nestin, and *SOX2*, respectively. (D-F and D’-F’) Double immunofluorescences profiling. (G’) The intra-neurospheral cells were co-positively expressed and localized nuclei markers which were characterized as NSCs (H) The fluorescent intensity. Data were expressed as the mean±SD; n=3, ***p<0.001. Scale bars: A-G and A’-G’ = 100 μm

Where it reads, page 10

**Figure 7-** Gene profiling. (a-c) The neurospheres presented the increasing expression of NSCs profiling (<i>NES, SOX1, </i>and <i>PAX6</i>) when compared to the hSCAPs. Data were expressed as the mean±SD; n=3, **<i>p </i><0.01, ***<i>p</i> <0.001

The sentence should read

**Figure 7-** Gene profiling. (A-C) The neurospheres presented the increasing expression of NSCs profiling (*NES*, *SOX1*, and *PAX6*) when compared to the hSCAPs. Data were expressed as the mean±SD; n=3, **p<0.01, ***p<0.001

Where it reads, page 11

**Figure 8-** Functionality test. (a) The hSCAPs weakly expressed fluorescent calcium ions signal. (b) The intra-neurospheral cells obviously revealed calcium ions signal. (c) The hSCAPs presented a low and narrow dynamic change of calcium ions intensity (red, orange, and yellow lines). Importantly, higher and wider dynamic changes of calcium ions intensity were observed at intra-neurospheral cells (pink, dark blue, and light blue lines). Data were expressed as the mean intensity of calcium ions; n=3

The sentence should read

**Figure 8-** Functionality test. (A-D) The hSCAPs weakly expressed fluorescent calcium ions signal. (A’-D’) The intra-neurospheral cells obviously revealed calcium ions signal. (A’’-D’’) The hSCAPs presented a low and narrow dynamic change of calcium ions intensity (red, orange, and yellow lines). Importantly, higher and wider dynamic changes of calcium ions intensity were observed at intra-neurospheral cells (pink, dark blue, and light blue lines). Data were expressed as the mean intensity of calcium ions; n=3, Scale bar: A-D and A’-D’ = 100 μm

